# Derivations of the Core Functions of the Maximum Entropy Theory of Ecology

**DOI:** 10.3390/e21070712

**Published:** 2019-07-21

**Authors:** Alexander B. Brummer, Erica A. Newman

**Affiliations:** 1 Department of Biomathematics, David Geffen School of Medicine, University of California, Los Angeles, CA 90095, USA; 2 Department of Ecology and Evolutionary Biology, University of California, Los Angeles, CA 90095, USA; 3 Department of Ecology and Evolutionary Biology, University of Arizona, Tucson, AZ 85721, USA

**Keywords:** information entropy, information theoretics, macroecology, metabolic theory, scaling, species abundance distribution, species-area relationship

## Abstract

The Maximum Entropy Theory of Ecology (METE), is a theoretical framework of macroecology that makes a variety of realistic ecological predictions about how species richness, abundance of species, metabolic rate distributions, and spatial aggregation of species interrelate in a given region. In the METE framework, “ecological state variables” (representing total area, total species richness, total abundance, and total metabolic energy) describe macroecological properties of an ecosystem. METE incorporates these state variables into constraints on underlying probability distributions. The method of Lagrange multipliers and maximization of information entropy (MaxEnt) lead to predicted functional forms of distributions of interest. We demonstrate how information entropy is maximized for the general case of a distribution, which has empirical information that provides constraints on the overall predictions. We then show how METE’s two core functions are derived. These functions, called the “Spatial Structure Function” and the “Ecosystem Structure Function” are the core pieces of the theory, from which all the predictions of METE follow (including the Species Area Relationship, the Species Abundance Distribution, and various metabolic distributions). Primarily, we consider the discrete distributions predicted by METE. We also explore the parameter space defined by the METE’s state variables and Lagrange multipliers. We aim to provide a comprehensive resource for ecologists who want to understand the derivations and assumptions of the basic mathematical structure of METE.

## 1. The Maximum Entropy Theory of Ecology

Many of the central questions of macroecology ask how patterns of species richness, abundance, and body size arise from ecosystems, how these patterns scale over increasing area, and how they interrelate [1]. Many macroecological distributions that quantify aspects of community structure, such as the Species–Area Relationship, the Species Abundance Distribution [2], size–density relationships [3,4,5], and the allometric scaling of metabolic rates of biological organisms within a community [6,7,8] have been studied independently, revealing general properties that may be universal across ecosystems. The Maximum Entropy Theory of Ecology (METE) [9,10,11], is a theoretical framework of macroecology that makes a variety of realistic ecological predictions about the diversity and structure of ecological communities [12,13,14,15,16,17,18,19,20]. These predictions relate species richness and abundance to metabolic rate distributions and spatial aggregation of species in a given region. Because METE makes a set of interrelated predictions about community structure, it has the potential to unify disparate parts of macroecology into a single mathematical framework.

The underlying mathematics of METE relies on a method termed “MaxEnt”: the maximization of information entropy. MaxEnt uses the method of Lagrange multipliers to find probability distributions that underlie statistical phenomena. For METE, the MaxEnt method is applied to problems involving measurable “ecological state variables” that describe macroecological properties of an ecosystem (Section 3.1). METE is a “top-down” theory that makes no assumptions regarding the particular details of, or mechanistic interactions between, the species being studied. This is different from other “bottom-up” theories involving the combinatorics of micro-states that result in Boltzmann distributions [21,22,23]. As such, its focus is the maximization of the Shannon information entropy [24,25].

In this paper, we will first demonstrate how information entropy is maximized for the general case of a distribution, which has empirical information that provides constraints on the overall predictions. We then introduce the ecological state variables $A_{0}$, $S_{0}$, $N_{0}$, and $E_{0}$, representing total area, total number of species, total abundance, and total metabolic energy of an ecological system, and use them with the method of information entropy maximization to show how METE’s two core functions are derived. These functions, called the “Spatial Structure Function” and the “Ecosystem Structure Function” are the core pieces of the theory, from which all the predictions of METE follow (including the Species–Area Relationship, the Species Abundance Distribution, and various metabolic distributions). Primarily, we consider the discrete distributions predicted by METE. These derivations are not provided in their entirety in Harte (2011) [10], but are the derivations that will produce the core distributions of the Spatial Structure Function and the Ecosystem Structure Function as presented in that work.

In the process of constructing the core structure functions, we derive the Lagrange multipliers that arise from the MaxEnt process, and characterize the ecosystems modeled by METE. We investigate the parameter space of these Lagrange multipliers, and evaluate some of the simplifying assumptions that have been used previously to estimate the Lagrange multiplier values.

We aim to provide a comprehensive resource for ecologists who want to understand the derivations and assumptions of the basic structure of METE. We hope that by providing explicit derivations of METE, we will encourage other ecologists to modify the framework, apply it to their own systems, and make progress in this valuable area of research.

## 2. Information Entropy Maximization: A Primer

In this section we present the equations that are necessary for information entropy maximization. We then use these equations to derive the form of the probability distribution resulting from the simplest case of a discrete, one-dimensional distribution. Although this derivation will not be new for regular readers of *Entropy*, we include it for ecologists who are interested in this theoretical framework, because this chain of logic will be applied to the constraints that characterize METE in subsequent sections.

### 2.1. Writing Down the Constraints

As observed by Haegeman and Etienne (2010) [26], probability distributions with higher information entropy encode less information. Therefore, a probability distribution that corresponds to empirical data without imputing any additional information will maximize information entropy. This is also true of a probability distribution that conforms to a constraint (as, for example, a constraint on the value of the mean) without making additional assumptions or adding other information. In this sense, maximum information entropy methods give the most impartial estimate of the shape of the underlying probability distribution for an observable. MaxEnt also gives the least biased estimators of the moments of a distribution (which include the range, mean, and variance) [25], meaning that there is no difference, for example, between the estimated mean and the empirical mean of a distribution. This feature of MaxEnt is by design, such that the moments of a probability distribution are constrained by the empirical values of those moments.

Here we present the primary equations that regularly occur in information entropy maximization. The general expression for *K* constraints on the mean values of the variables $f_{k}\left(n\right)$, where *n* follows the distribution p(n), is expressed as:

(1) $$\sum_{n=1}^{n=N}f_{k}\left(n\right)p\left(n\right)=\langle f_{k}\rangle .$$

An additional constraint provides for the normalization of the probability distributions, and is expressed as:

(2) $$\sum_{n=1}^{n=N}p\left(n\right)=1.$$

The procedure of maximizing entropy results in a particular form of the probability distribution and partition function, proven by Jaynes [25] to result in the least-biased probability p(n) that satisfies the constraint equation of Equation (1) and the normalization constraint of Equation (2),

(3) $$p\left(n\right)=\frac{1}{Z}\exp\left\{-\sum_{k=1}^{k=K}\lambda_{k}f_{k}\left(n\right)\right\},$$

where *Z* is the partition function that serves to normalize the probability distribution, and is expressed as,

(4) $$Z=\sum_{n=1}^{n=N}\exp\left\{-\sum_{k=1}^{k=K}\lambda_{k}f_{k}\left(n\right)\right\}.$$

Generally, when one wants to use the tools of MaxEnt, one will need to have data from which constraints on the distributions can be inferred (such as average values). Then a process of algebra and numerical methods will begin during which one solves for the Lagrange multipliers $\lambda_{k}$. Once the Lagrange multipliers have been determined, then the form of the probability distributions can be inferred (or graphed). For examples using Equations (1)–(4), see Appendix A.1. Equations (3) and (4) are written here for reference, as we will use them repeatedly. In the next section we derive Equations (3) and (4) by maximizing the Shannon information entropy.

### 2.2. The Method of Lagrange Multipliers and Optimization

Here we derive the generic probability distribution p(n) for the specific scenario of a discrete variable with one constraint (beyond normalization). This will serve as a simpler example for deriving the core distributions, or structure functions of METE.

What does it mean for us to “maximize Shannon information entropy”? While the explicit form of the probability distribution, p(n), is unknown, we have information about some of its properties that will serve to constrain its functional form. However, there may exist more than one function p(n) that satisfies our constraints, so we must choose between them. By maximizing Shannon information entropy we are maximizing the uncertainty inherent in our function p(n), and choosing the unique form of p(n) that represents the least amount of bias in regard to our measurements. The expression for Shannon information entropy [24] is:

(5) $$H=-\sum_{n=N_{min}}^{N_{max}}p\left(n\right)\ln\left(p\left(n\right)\right).$$

We know that no matter what the form of the probability distribution is, it should be properly normalized (that is, the sum of the probabilities of all possible outcomes will equal one). This gives us our normalization constraint, which for a single, discrete variable takes the form of

(6) $$\sum_{n=N_{min}}^{N_{max}}p\left(n\right)=1.$$

Finally, we will likely have some information about an aggregated measurement of the variable in our system. This information constitutes our additional constraint, or constraints. Letting f(n) represent a measured value dependent on *n*, and assuming that the aggregated measurement we have is the mean value of f(n), then we can express our knowledge regarding this measurement of an observable quantity that represents some aggregated or average value using the mathematical definition of an average.

(7) $$\sum_{n=N_{min}}^{N_{max}}f\left(n\right)p\left(n\right)=\langle f\rangle .$$

Thus, the definition itself constitutes our constraint.

To maximize Shannon information entropy subject to our constraints, we employ the tools of variational calculus and the method of undetermined Lagrange multipliers. A concrete example of this is given in Appendix A.1. We begin by constructing the function F, which is an expression that incorporates the measure of Shannon information entropy and the additional constraints.

(8) $$F=-\sum_{n=N_{min}}^{N_{max}}p\left(n\right)\ln\left(p\left(n\right)\right)-\lambda_{0}\left[\sum_{n=N_{min}}^{N_{max}}p\left(n\right)-1\right]-\lambda_{1}\left[\sum_{n=N_{min}}^{N_{max}}f\left(n\right)p\left(n\right)-\langle f\rangle \right].$$

The constraints are written in such a way so that each constraint is independent of the other, and each term inside the square brackets is zero. In this way, we can incorporate multiple constraints without changing the overall value of F. When we perform the maximization step to find local optima, the presence of the constraints will change the subsequent form of p(n). That is, maximizing F will subsequently maximize the uncertainty represented by the Shannon information entropy, *H*, subject to the constraints. Thus we seek to solve for the functional form of p(n) that results from variations in F, due to variations in p(n). Mathematically this is expressed as δF/δp(n)=0. Practically, this requires evaluating derivatives of each term in F with respect to p(n), and setting the resulting equation equal to zero,

(9) $$0=-\left[\ln(p(n))+1\right]-\lambda_{0}-\lambda_{1}f\left(n\right).$$

Note that we can drop the summations at this point. Solving for p(n) yields,

(10) $$p\left(n\right)=k\exp\left\{-\lambda_{1}f\left(n\right)\right\},$$

where $k=\exp\left\{-(\lambda_{0}+1)\right\}$. Imposing our normalization constraint $\sum_{n}p\left(n\right)=1$, we have

(11) $$\sum_{n}k\exp\left\{-\lambda_{1}f\left(n\right)\right\}=1.$$

Since *k* is independent of *n*, we can factor it out of the summation and solve for it to find,

(12) $$k=\frac{1}{\sum_{n}\exp\left\{-\lambda_{1}f\left(n\right)\right\}}.$$

The expression $\sum_{n}\exp\left\{\lambda_{1}f\left(n\right)\right\}$ comes up so often that it is given its own variable representation *Z*, the partition function, which will eventually just turn out to be a real-valued number,

(13) $$Z=\sum_{n}\exp\left\{-\lambda_{1}f\left(n\right)\right\}.$$

Thus, we can express the probability p(n) as,

(14) $$p\left(n\right)=\frac{1}{Z}\exp\left\{-\lambda_{1}f\left(n\right)\right\}=\frac{\exp\left\{-\lambda_{1}f\left(n\right)\right\}}{\sum_{n}\exp\left\{-\lambda_{1}f\left(n\right)\right\}}.$$

To find the explicit form of p(n), one needs further information in the form of data. Having data, it is then possible to use the constraint equations to numerically solve for the undetermined Lagrange multipliers, and thus identify the form of the probability distribution p(n). This relationship between data, the Lagrange multipliers, and the resulting form of the probability distribution is revisited for normal and log-normal distributions in Appendix A.3 and Appendix A.4, and is fundamental to the Maximum Entropy Theory of Ecology.

## 3. The Structure of METE

In this section, we introduce and discuss the ecological state variables common to macroecology, as used by METE. We apply the MaxEnt method to these ecological state variables to derive the core distributions of METE, namely the Spatial Structure Function, and the Ecosystem Structure Function [10,11].

### 3.1. A State Variable Theory

Much of macroecology is concerned with detecting patterns in ecosystems, either at large scale, or as an emergent property of scaling over larger aggregates of individuals, species, area, or time [27]. To detect these patterns, we must work with variables that are sufficiently coarse that they capture average conditions of some larger phenomena that is being modeled, or sufficiently aggregated that they apply across systems and capture certain generalities. For example, we may examine average abundance of all individuals in an area. The “average” may refer to an average of repeated measurements in different plots or subplots, for example, which can smooth out heterogeneity and model average conditions on a landscape. The “aggregated” nature of abundance refers to something slightly different, in that abundance measured will be a result of multiple processes, such as birth, death, emigration, and immigration [28]. Abundance therefore represents an “aggregate” metric of all of these processes. Similarly, “species” as an observable may have an average value when measured in many similar sized plots, but is itself an aggregate measure of biodiversity that ignores (or “coarse grains”) genetic variation among individuals, and processes such as hybridization. These variables are easily measured and compared across ecological systems, and have an overall generality and transferability that make them relevant to ecological studies at large scales.

Within METE, variables representing total area, total number of species, total abundance, and total metabolic energy of an ecological system are central to the definitions of the core probability distributions. These ecological state variables are represented as $A_{0}$, $S_{0}$, $N_{0}$, and $E_{0}$, respectively. They are static (not time-dependent), and can be used to model macroecological distributions of interest, such as the Species–Area Relationship, the Species Abundance Distribution, and various metabolic rate distributions, both within a species and across an entire community. This so-called “ASNE” version of METE, where “ASNE" represents the four state variables above, has been the subject of the most study [10], but other constraints featuring additional state variables are possible. In one case, METE has been extended to include higher taxonomic constraints [29].

It is worth clarifying that although “ecological state variables” may call to mind the true state variables of thermodynamics, the use of this term is merely an analogy to thermodynamics concepts [30], but differs in underlying justification and interpretation. Ecological state variables capture information that defines the state of the system at a given point in time and space, but cannot be interpreted as true macroscopic variables for many reasons, including the less-than-ideal behavior of the variables themselves. For example, $S_{0}$, the total number of species in an area, neither adds nor averages when considering larger and larger areas. It is therefore neither truly an intensive nor an extensive variable. “Species” as a defined concept only applies to a fraction of real organisms [31], and is used in macroecology as an aggregating variable, either sorting organisms by their actual species designation, or as a stand-in for phylogenetic relatedness or functional traits [32]. Because of these and other issues, ecological state variables are seen to embody information at an aggregate level, and are not interpreted as direct translation from countable microstates to macrostates, though this has been the subject of some debate [30,33,34,35]. This raises a related issue, which is the use of the Shannon form of entropy in METE and the information-entropy interpretation.

Work with applications of entropy maximization to macroecological patterns began in 2006 with Shipley, Vile, and Garnier’s use of Shannon information entropy in the problem of constructing Species Abundance Distributions [33]. This advance sparked a small explosion of the use of entropy maximization in macroecology, including applications to new problems (variously using frequentist and Bayesian interpretations of MaxEnt), a search for generalities among ecosystems, and among the approaches themselves [9,13,14,17,36,37,38,39,40,41,42,43,44,45]. It also sparked a debate about the use of information entropy in macroecology, in which Shipley [30,33] and Haegeman and Loreau [34,35], discuss the use and justification of Shannon information entropy in ecology. In this exchange, Shipley maintains that ecological systems are more complex in their constraints than systems of particles, and that we may never know (or be able to measure) all of the relevant constraints. He therefore suggests focusing on the most important constraints, and using information entropy to evaluate the constraints and distributions. Although Haegeman and Loreau make a clear argument that the frequentist interpretation of MaxEnt should be complementary to the information entropy interpretation, Shipley disagreed, arguing incorporating all relevant constraints may not be possible for ecological systems, but would be needed for the constraint-based information entropy and state-counting, combinatorics interpretations of entropy to be equivalent. A later exchange between Favretti [21,23] and Harte [22] revisits this topic, and more productive work may be done to address the issues raised in all of these exchanges.

METE follows the logic of Shipley’s arguments about the use of important constraints in complex ecosystems, and uses the information entropy approach. In the following sections, we will demonstrate how METE’s ecological state variables and their ratios are used with the MaxEnt procedure to constrain patterns of individuals over area for the Spatial Structure Function, and patterns of metabolic requirement (or body size) across individuals and abundances per species through the Ecosystem Structure Function.

### 3.2. The Spatial Structure Function

This distribution goes by multiple names among practitioners, including the “Pi distribution” (informally), the “Species-level Spatial Abundance Distribution” (SSAD) [14,46], and the Spatial Structure Function (SSF) [47], which is how we will refer to it here. The SSF is a by-species prediction of the clustering of individuals over space, and is defined as the “probability that *n* individuals of a species are found in a cell of area *A* if it has $n_{0}$ individuals in $A_{0}$” [10]. We derive this distribution before the Ecosystem Structure Function for pedagogical reasons, as it is a one-dimensional distribution for a discrete variable. This variable is the the abundance of a single species, *n*, at a given scale, *A*, where *A* is a smaller area within the total area under consideration, $A_{0}$. The variables that are used to constrain the system are *A*, $A_{0}$, and the abundance of a single species at the total spatial scale, $n_{0}$. The derivation of the Spatial Structure Function will follow exactly the general approach provided in the previous section. We represent this function as $\Pi (n|A,n_{0},A_{0})$. To ensure that the Spatial Structure Function is properly normalized, we express our normalization constraint as,

(15) $$\sum_{n=0}^{n_{0}}\Pi \left(n\right)=1.$$

where we have dropped the conditional variables $A,n_{0}$, and $A_{0}$ for notational convenience. Note also that the lower limit on the summation is set to n=0. This is because *n* represents the per-species abundance, and it is possible for a species that has $n_{0}$ individuals to have zero abundance in an area *A*.

Our additional constraint comes from the simultaneous definition and measurement of the average value of the per-species abundance $\bar{n}$. From measurement, $\bar{n}=n_{0}A/A_{0}$. From definition, $\bar{n}=\sum_{n=0}^{n_{0}}n\Pi \left(n\right)$. Combining these two expressions gives us our other constraint,

(16) $$\sum_{n=0}^{n_{0}}n\Pi \left(n\right)=\frac{n_{0}A}{A_{0}}.$$

Now, as we want to maximize uncertainty, or Shannon information entropy, related to the Spatial Structure Function subject to the above two constraints, we construct the function F in the form,

(17) $$F=-\sum_{n=0}^{n_{0}}\Pi \left(n\right)\ln\left(\Pi \left(n\right)\right)-\lambda_{0}\left[\sum_{n=0}^{n_{0}}\Pi \left(n\right)-1\right]-\lambda_{\Pi }\left[\sum_{n=0}^{n_{0}}n\Pi \left(n\right)-\frac{n_{0}A}{A_{0}}\right].$$

From here we maximize F by evaluating the expression δF/δΠ(n)=0. This results in,

(18) $$0=-\left[\ln(\Pi (n))+1\right]-\lambda_{0}\left(1\right)-\lambda_{\Pi }n.$$

From here we can solve for Π(n) to arrive at,

(19) $$\Pi \left(n\right)=k\exp\left\{-\lambda_{\Pi }n\right\}$$

where $k=\exp\{-\left(\lambda_{0}+1\right)\}$. Imposing our normalization constraint, we can write,

(20) $$\sum_{n=0}^{n_{0}}k\exp\left\{-\lambda_{k}n\right\}=1.$$

Because *k* is independent of *n*, we can factor it out of the summation and rewrite as,

(21) $$k=\frac{1}{\sum_{n=0}^{n_{0}}\exp\left\{-\lambda_{\Pi }n\right\}}.$$

Conventionally this normalization constant is defined as 1/Z and denoted as the partition function, where,

(22) $$Z=\sum_{n=0}^{n_{0}}\exp\left\{-\lambda_{\Pi }n\right\}.$$

This brings us to the following compact expression for the Spatial Structure Function,

(23) $$\Pi \left(n\right)=\frac{1}{Z}\exp\left\{-\lambda_{\Pi }n\right\}.$$

In order to actually calculate, or graph, the Spatial Structure Function for a given set of values $A_{0},n_{0},$ and *A*, we must first calculate the Lagrange multiplier $\lambda_{\Pi }$ using our constraint equation relating the measured average per-species abundance $\bar{n}=n_{0}A/A_{0}$ to the definition of the average per-species abundance $\bar{n}=\sum_{n=0}^{n_{0}}n\Pi \left(n\right)$. This time, substituting our known expression for Π(n), we have,

(24) $$\sum_{n=0}^{n_{0}}\frac{n}{Z}\exp\left\{-\lambda_{\Pi }n\right\}=\frac{n_{0}A}{A_{0}}.$$

Recalling that *Z* is independent of *n*, it can be pulled out of the summation. Furthermore, substituting our definition of *Z*, but using *m* as a dummy index instead of *n* to avoid mixing up our indices, we can write,

(25) $$\frac{\sum_{n=0}^{n_{0}}n\exp\left\{-\lambda_{\Pi }n\right\}}{\sum_{m=0}^{n_{0}}\exp\left\{-\lambda_{\Pi }m\right\}}=\frac{n_{0}A}{A_{0}}.$$

**Figure 1:**
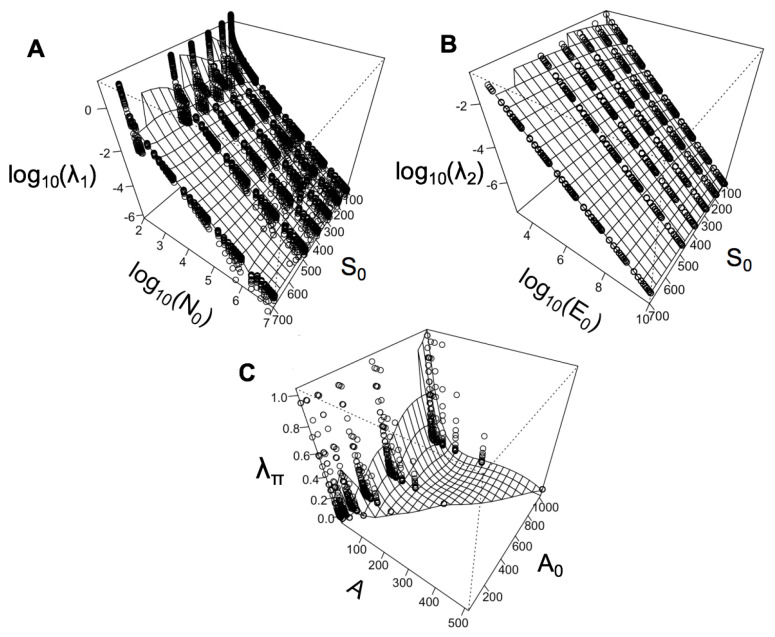
The relationship between the Maximum Entropy Theory of Ecology (METE)’s Lagrange multipliers $\lambda_{1}$, $\lambda_{2}$, and $\lambda_{\Pi }$, and the ecological state variables in the mathematical constraints that produce them. Values for each λ were generated with the software package meteR [47] in the statistical computing language R [48], and a surface was interpolated to aid in visualization. In panel (**A**), we see the greater influence of log($N_{0}$) than $S_{0}$ on the overall value of the Lagrange multiplier $\lambda_{1}$, and a compression of $\lambda_{1}$ values at low $N_{0}$. In panel (**B**), we can see a near-linear relationship on the log-log scale between $\lambda_{2}$ and log($E_{0}$), while $S_{0}$ does not affect its value as greatly over this range of values. In panel (**C**), we see a highly non-linear relationship between $\lambda_{\Pi }$, the state variable $A_{0}$, and the smaller area under consideration, *A*.

In principle, the above expression allows one to solve for the Lagrange multiplier $\lambda_{\Pi }$. However, analytical solutions for $\lambda_{\Pi }$ are intractable, thus one must resort to numerical methods. We graph the parameter space of the state variables $A_{0},n_{0},n$ and $\lambda_{\Pi }$ in Figure 1.

### 3.3. The Ecosystem Structure Function

The Ecosystem Structure Function (ESF) is the second of METE’s core distributions. Unlike the SSF, it does not have a simple definition, but can be described as a kind of “container function” that describes the probability space, R(n,ϵ), of how abundances are assigned to species, *n*, and how metabolic energy, ϵ, is partitioned over individuals in a community. As described by Bertram (2015) [49], R(n,ϵ) is a joint probability distribution, with R(n,ϵ)dϵ by definition being “the probability that a randomly selected species has abundance *n*, and that a randomly selected individual from any species with abundance *n* has metabolic requirement in the interval (ϵ,ϵ+dϵ)” (page 55). This definition differs from and corrects the one given in Harte (2011) [10] (also used in [45]), and is consistent with a later correction to the Harte (2011) definition [21] (accepted by Harte [22]). The ESF is the distribution from which the Species Abundance Distribution, Species–Area Relationship, and metabolic rate distributions [14,18] can eventually be derived. Constrained by empirical values measured from real systems, the ESF produces actual predictions of these probability distributions.

As the ESF R(n,ϵ), depends on one discrete variable *n*, and one continuous variable ϵ, we will this time need to integrate over ϵ in addition to summing over *n*. Thus, our normalization constraint now takes the form of,

(26) $$\sum_{n=1}^{N_{0}}\int_{\epsilon =1}^{E_{0}}R\left(n,\epsilon \right)d\epsilon =1.$$

Note that in the above expression we have specified the lower limit of metabolism as, $\epsilon_{min}=1$. In so doing we have defined metabolism as a dimensionless quantity. This decision has several consequences. For comparison against data, a researcher must standardize their metabolic measurements to conform with this definition. That is, they divide all metabolic measurements by the smallest measured value [10,14,19]. A second consequence of this decision is that it preempts the need to incorporate reference distributions when expressing Shannon information entropy for a continuous variable [10,25].

Our additional constraints are aggregated measures of variables *n* and nϵ, that is $f_{1}\left(n\right)=n$, and $f_{2}\left(n\epsilon \right)=n\epsilon$. The measures themselves are the ratios $N_{0}/S_{0}$ and $E_{0}/S_{0}$, that is $\langle f_{1}\left(n\right)\rangle =N_{0}/S_{0}$, and $\langle f_{2}\left(n\epsilon \right)\rangle =E_{0}/S_{0}$. This gives us the pair of constraints,

(27) $$\begin{array}{c} \sum_{n=1}^{N_{0}}\int_{\epsilon =1}^{E_{0}}nR\left(n,\epsilon \right)d\epsilon =\frac{N_{0}}{S_{0}} \end{array}$$

(28) $$\begin{array}{c} \sum_{n=1}^{N_{0}}\int_{\epsilon =1}^{E_{0}}n\epsilon R\left(n,\epsilon \right)d\epsilon =\frac{E_{0}}{S_{0}}. \end{array}$$

Now, the function F that we will want to maximize takes the form of

$$\begin{array}{ccc} F & = & -\sum_{n=1}^{N_{0}}\int_{\epsilon =1}^{E_{0}}R\left(n,\epsilon \right)\ln\left(R\left(n,\epsilon \right)\right)d\epsilon -\lambda_{0}\left[\sum_{n=1}^{N_{0}}\int_{\epsilon =1}^{E_{0}}R\left(n,\epsilon \right)d\epsilon -1\right]\\ & - & \lambda_{1}\left[\sum_{n=1}^{N_{0}}\int_{\epsilon =1}^{E_{0}}nR\left(n,\epsilon \right)d\epsilon -\frac{N_{0}}{S_{0}}\right]-\lambda_{2}\left[\sum_{n=1}^{N_{0}}\int_{\epsilon =1}^{E_{0}}n\epsilon R\left(n,\epsilon \right)d\epsilon -\frac{E_{0}}{S_{0}}\right]. \end{array}$$

As before, we maximize F by evaluating δF/δR=0. This results in,

(29) $$0=-\left[\ln(R(n,\epsilon ))+1\right]-\lambda_{0}\left[1\right]-\lambda_{1}\left[n\right]-\lambda_{2}\left[n\epsilon \right].$$

Solving for R(n,ϵ) gives,

(30) $$R\left(n,\epsilon \right)=k\exp\left\{-\lambda_{1}n-\lambda_{2}n\epsilon \right\},$$

where $k=\exp\{-\left(1+\lambda_{0}\right)\}$. Using our normalization condition to define *Z* we have,

(31) $$Z=\sum_{n=1}^{N_{0}}\int_{\epsilon =1}^{E_{0}}\exp\left\{-\lambda_{1}n-\lambda_{2}n\epsilon \right\}d\epsilon .$$

This allows us to express the full form of the Ecosystem Structure Function as,

(32) $$R\left(n,\epsilon \right)=\frac{\exp\{-\lambda_{1}n-\lambda_{2}n\epsilon \}}{\sum_{m=1}^{N_{0}}\int_{\epsilon^{\prime }=1}^{E_{0}}\exp\left\{-\lambda_{1}m-\lambda_{2}m\epsilon^{\prime }\right\}d\epsilon^{\prime }},$$

where we have replaced *n* with *m* and ϵ and $\epsilon^{\prime }$ in the denominator to ensure there is no confusion over which variables belong in the numerator or denominator.

At this point we can simplify the expression for *Z* by performing the integral over $\epsilon^{\prime }$. Factoring out the term in *Z* independent of $\epsilon^{\prime }$ we have,

(33) $$Z=\sum_{m=1}^{N_{0}}\exp\left\{-\lambda_{1}m\right\}\int_{\epsilon^{\prime }=1}^{E_{0}}\exp\left\{-\lambda_{2}m\epsilon^{\prime }\right\}d\epsilon^{\prime }.$$

After integrating, we have

(34) $$Z=\sum_{m=1}^{N_{0}}\frac{\exp\{-\lambda_{1}m\}}{\lambda_{2}m}\left[\exp\left\{-\lambda_{2}m\right\}-\exp\left\{\lambda_{2}mE_{0}\right\}\right],$$

and with further simplification,

(35) $$Z=\frac{1}{\lambda_{2}}\sum_{m=1}^{N_{0}}\frac{1}{m}\left[\exp\{-m\beta \}-\exp\{-m\sigma \}\right],$$

where $\beta =\lambda_{1}+\lambda_{2}$ and $\sigma =\lambda_{1}+E_{0}\lambda_{2}$.

From here we can examine the constraint Equations (27) and (28). In particular, now that we have an explicit form for the structure function, we can perform the integrals in the constraint equations to fix the values of the unknown Lagrange multipliers in terms of the measured quantities $N_{0},E_{0},$ and $S_{0}$. Upon substitution of our expression for R(n,ϵ) into Equation (27) we have,

(36) $$\frac{N_{0}}{S_{0}}=\frac{1}{Z}\sum_{n=1}^{N_{0}}\int_{\epsilon =1}^{E_{0}}n\exp\left\{-n\left(\lambda_{1}+\lambda_{2}\epsilon \right)\right\}d\epsilon .$$

Factoring from the integral the term independent of ϵ,

(37) $$\frac{N_{0}}{S_{0}}=\frac{1}{Z}\sum_{n=1}^{N_{0}}n\exp\left\{-n\lambda_{1}\right\}\int_{\epsilon =1}^{E_{0}}\exp\left\{-n\lambda_{2}\epsilon \right\}d\epsilon .$$

Upon integration,

(38) $$\frac{N_{0}}{S_{0}}=\frac{1}{Z}\sum_{n=1}^{N_{0}}n\exp\left\{-n\lambda_{1}\right\}\left[\frac{\exp\left\{-n\lambda_{2}\right\}-\exp\left\{-n\lambda_{2}E_{0}\right\}}{n\lambda_{2}}\right],$$

and with further simplification,

(39) $$\frac{N_{0}}{S_{0}}=\frac{1}{Z\lambda_{2}}\sum_{n=1}^{N_{0}}\left[\exp\{-n\beta \}-\exp\{-n\sigma \}\right],$$

where $\beta =\lambda_{1}+\lambda_{2}$ and $\sigma =\lambda_{1}+E_{0}\lambda_{2}$.

Turning our attention now to constraint Equation (28), upon substitution of R(n,ϵ) we have,

(40) $$\frac{E_{0}}{S_{0}}=\frac{1}{Z}\sum_{n=1}^{N_{0}}\int_{\epsilon =1}^{E_{0}}n\epsilon \exp\left\{-n\left(\lambda_{1}+\lambda_{2}\epsilon \right)\right\}d\epsilon .$$

Factoring from the integral the term independent of ϵ,

(41) $$\frac{E_{0}}{S_{0}}=\frac{1}{Z}\sum_{n=1}^{N_{0}}n\exp\left\{-n\lambda_{1}\right\}\int_{\epsilon =1}^{E_{0}}\epsilon \exp\left\{-n\lambda_{2}\epsilon \right\}d\epsilon .$$

After integrating by parts we have,

(42) $$\frac{E_{0}}{S_{0}}=\frac{1}{Z}\sum_{n=1}^{N_{0}}n\exp\left\{-n\lambda_{1}\right\}\left[\frac{\exp\left\{-n\lambda_{2}\right\}-E_{0}\exp\left\{-n\lambda_{2}E_{0}\right\}}{n\lambda_{2}}+\frac{\exp\left\{-n\lambda_{2}\right\}-\exp\left\{-n\lambda_{2}E_{0}\right\}}{{\left(n\lambda_{2}\right)}^{2}}\right],$$

and with further simplification,

(43) $$\frac{E_{0}}{S_{0}}=\frac{1}{Z}\sum_{n=1}^{N_{0}}\left[\frac{\exp\left\{-n\beta \right\}-E_{0}\exp\left\{-n\sigma \right\}}{\lambda_{2}}+\frac{\exp\{-n\beta \}-\exp\{-n\sigma \}}{n\lambda_{2}^{2}}\right],$$

where $\beta =\lambda_{1}+\lambda_{2}$ and $\sigma =\lambda_{1}+E_{0}\lambda_{2}$. Substituting in our expression for *Z*, we can write the integrated versions of the constraint equations in full as,

(44) $$\begin{array}{ccc} \frac{N_{0}}{S_{0}} & = & \frac{\sum_{n=1}^{N_{0}}\left[\exp\{-n\beta \}-\exp\{-n\sigma \}\right]}{\sum_{m=1}^{N_{0}}\left[\frac{\exp\{-m\beta \}-\exp\{-m\sigma \}}{m}\right]} \end{array}$$

(45) $$\begin{array}{ccc} \frac{E_{0}}{S_{0}} & = & \frac{\sum_{n=1}^{N_{0}}\left[\exp\left\{-n\beta \right\}-E_{0}\exp\left\{-n\sigma \right\}\right]}{\sum_{m=1}^{N_{0}}\left[\frac{\exp\{-m\beta \}-\exp\{-m\sigma \}}{m}\right]}+\frac{1}{\lambda_{2}}. \end{array}$$

The values of $\lambda_{1}$ and $\lambda_{2}$ are often difficult to calculate by conventional means, so some approximations were used in Harte (2011). With the use of the software package meteR [47,48], which implements a number of METE’s predictions and tests them against empirical data sets, these approximations are no longer necessary. However, we consider them in more depth in Appendix B.

## 4. Relationships between State Variables and Lagrange Multipliers

By using the MaxEnt approach with the METE ecological state variables, we derive three Lagrange multipliers: $\lambda_{\Pi }$ associated with the Spatial Structure Function, and $\lambda_{1}$ and $\lambda_{2}$, associated with the Ecosystem Structure Function. The METE Lagrange multipliers represent all the possible relationships between $N_{0}$, $S_{0}$, and $E_{0}$, and METE’s predicted relationships of *n*, *A*, and $A_{0}$. We graph these, along with the ecological state variables in their respective constraints, in Figure 1.

Examining $\lambda_{1}$ in panel (A), we see the greater influence of log($N_{0}$) than $S_{0}$ on the overall value of the Lagrange multiplier $\lambda_{1}$, and a compression of $\lambda_{1}$ values at low $N_{0}$. This may correspond to METE’s own stated limitations, and its requirement that N>>1 [10]. It also implies that $\lambda_{1}$ may be quite sensitive to the total area being sampled in empirical studies, and that this factor should be explicitly controlled for when comparing diversity patterns, such as Species Abundance Distributions, across plots. In panel (B), we can see a near-linear relationship on the log-log scale between $\lambda_{2}$ and log($E_{0}$), while $S_{0}$ does not affect its value as greatly over this range of values. It is apparent, then, that the METE distributions that rely on $\lambda_{2}$, such as the species-level energy distributions [14,18] are driven by body size, and may be largely insensitive to species richness, which in turn implies that the metabolic predictions of METE should hold equally well in very different ecosystems that share size characteristics, such as boreal forests and tropical ones.

In panel (C), $\lambda_{\Pi }$ is clearly non-linear in both the state variable $A_{0}$, and the smaller area under consideration, *A*. The graph of $\lambda_{\Pi }$ shows that very different values of $\lambda_{\Pi }$ may be obtained by varying the ratio of *A* to $A_{0}$ slightly, and this may in turn suggest that it does not have the properties we would desire in a metric describing clustering. It has been demonstrated that the SSF does not always produce reliable predictions for clustering of individuals of a species within a a given area [16,46], and this area of METE could be extended and modified in future work. Further investigations of the relationships of $\lambda_{1}$ and $\lambda_{2}$ with data from varied ecosystems may also allow us to investigate patterns of diversity, abundance, body size, and the relationships between these macroecological variables in new ways. These kinds of parameter space representations are also useful in generating hypotheses about changing ecosystems, and what new distributions are expected as one or more state variable changes.

**Figure 2:**
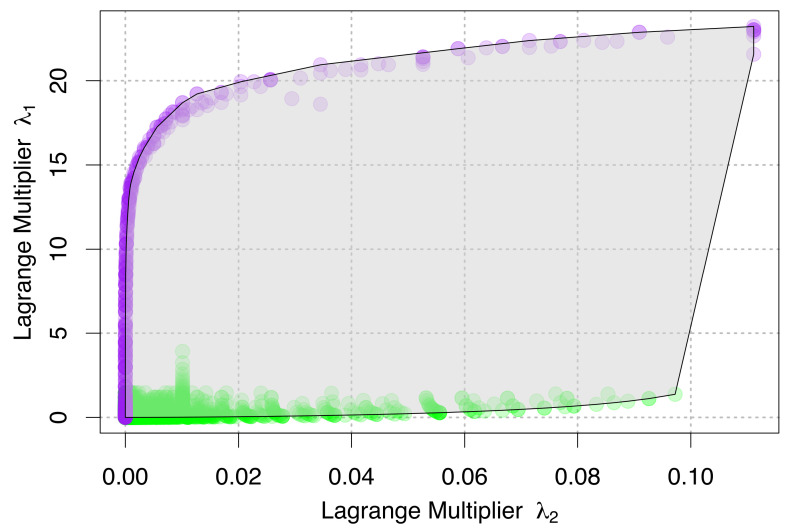
The parameter space of ecosystems as defined by the METE Lagrange multipliers $\lambda_{1}$, corresponding to the constraint on $\frac{N_{0}}{S_{0}}$, and $\lambda_{2}$, corresponding to the constraint on $\frac{E_{0}}{S_{0}}$. The highest values of $\lambda_{1}$ for any value of $\lambda_{2}$ correspond to values of $\frac{N_{0}}{S_{0}}$ = 1 (shown in purple), or situations where there is only one individual per species (small numbers of measurements or high diversity). Most real ecosystems and empirical systems with more than a few individuals are expected to fall closer to the low $\lambda_{1}$ values for any given $\lambda_{2}$ value (shown in green).

In Figure 2, we graph the parameter space that is defined by the ESF through the Lagrange multipliers $\lambda_{1}$ and $\lambda_{2}$. In this graph, the boundaries of the defined parameter space become interesting. High values of $\lambda_{1}$ always correspond to one or more “singleton species”, or species with a single individual, where $N_{0}$ = $S_{0}$. This can only happen when a single individual is measured in order to estimate the values of $A_{0}$, $N_{0}$, $S_{0}$, and $E_{0}$ (that is, small numbers of measurements), or in cases where there are the most species possible given the number of individuals present (extreme diversity). These cases therefore represent theoretical limits of possible outcomes of measurement. We expect that most real systems will have many measurements of species with more than one individual, and will fall into the range of low $\lambda_{1}$ values. The behavior of the lower values of the graph may therefore be worth investigating further in relation to spatial scale. Comparisons to empirical data sets may yield new, emergent patterns in this parameter space.

## 5. Summary

In this paper, we provide the explicit chain of logic that produces the core structure functions of Maximum Entropy Theory of Ecology, or METE. These derivations fill a gap in the ecological literature for researchers who would like to see the explicit construction and assumptions of the central equations from which the predictions of METE follow. In presenting this theory, we take a different pedagogical approach than is employed in Harte (2011) [10]. Namely, we provide a general case of a discrete MaxEnt problem in one dimension. We then give worked examples of constraints on moments beyond just the mean of a distribution (in Appendix A). From there, we first construct the simpler Spatial Structure Function, which is a discrete probability distribution, and then work through the logic of the Ecosystem Structure Function, which contains more constraints, and has a combination of discrete and continuous variables.

We do not cover the applications of METE to testing theory against empirical data, which has been done extensively elsewhere [9,10,11,12,13,14,15,16,17,18,19,20], however, we explicitly show the MaxEnt process and how it is applied to the ecological state variables of METE to produce the core structure functions from which all the predictions of METE derive.

Finally, we discuss the three Lagrange multipliers that result from applying the MaxEnt procedure to METE’s ecological state variables, and how these Lagrange multipliers characterize the system being studied. We investigate (in Appendix B) some of the simplifying approximations that were previously used to evaluate the Lagrange multipliers and assess their realism.

The equations presented here should make it easier for other researchers to make advances in MaxEnt-based macroecology, and METE in particular, either by investigating new state variables, or new functional forms of the mathematical constraints, and alternate forms of the entropy function. The Lagrange multipliers, graphed for the first time here, form a parameter space that may be useful to further studies involving comparisons between ecosystems and across scales.

## Appendix A. Examples of Applying MaxEnt to Known Distributions

Below we include basic equations for the application of MaxEnt to simple problems. This material is included for pedagogical value, and we show it for context. Although regular readers of *Entropy* may already be familiar with this material and versions available in textbooks (e.g., [50], Chapter 2), we are not aware of any ecological textbooks that contain this information. Readers who come from an ecology background may therefore not be familiar with these equations or approaches.

### Appendix A.1. A Fair Three-Sided Die Constrained by the Mean

Consider the case of a three-sided die with sides labeled 1, 2, and 3. Suppose we perform an experiment (that is, rolling a die), and measure the result (that is, reading and recording the outcome of the roll in the form of the number on the face-up side). After performing enough of these experiments, we can determine that the long-term average value of the rolls is to equal to two, by adding up all the outcomes and then dividing by the number of rolls. We take note that this is also the true average of a fair die, where all outcomes are equally probable. Because we chose a fair die in the first place, we are not surprised that our empirical outcome matches the outcome for a fair die. However, we are interested in determining the probability distribution that is associated with rolling any die, fair or not. This mathematical approach will allow us to find our empirical average and figure out what the probabilities of each outcome are, whether or not we have a fair die. To work through the logic, we will use the tools presented in the form of equations Equations (1), (3) and (4).

First, we start by writing Equation (1), and substituting in the values corresponding to our problem. Note that we have only one constraint (knowledge of the empirical mean), so K=1, and all sums over *k* vanish. Thus, $f_{k}\left(n\right)$ becomes f(n). Next, we can only make three possible observations, corresponding to the values on the three sides of the dice. So f(1)=1,f(2)=2, and f(3)=3. Finally, assuming that we have performed this experiment and have a mean value for f(n) to calculate from the possible observations, we can write that 〈f(n)〉=(3+2+1)/3=2. So, Equation (1) becomes,

(A1) 1p(1)+2p(2)+3p(3)=2.

This can be read as “the sum of the probabilities of a particular outcome of a roll times the value of that roll equals the empirical average of all outcomes on repeated trials”. Next, we can substitute our problem-specific values into the definitions of the probability distribution p(n) and the partition function *Z*. Doing so makes Equation (3) take on the form:

(A2) $$p\left(n\right)=\frac{1}{Z}e^{-\lambda n}$$

for generic values of *n*, or specifically,

(A3) $$\begin{array}{cc} p(1) & =\frac{1}{Z}e^{-\lambda }\\ p(2) & =\frac{1}{Z}e^{-\lambda 2}\\ p(3) & =\frac{1}{Z}e^{-\lambda 3} \end{array}$$

We have one Lagrange multiplier, here denoted as λ, and the probability of each outcome is expressed in terms of the partition function, as well as a term that involves the actual value of the outcome multiplied by the Lagrange multiplier. The partition function, which is defined in Equation (4), takes on the form:

(A4) $$\begin{array}{cc} Z & =\sum_{n=1}^{n=3}e^{-\lambda n}\\ Z & =e^{-\lambda }+e^{-\lambda 2}+e^{-\lambda 3}. \end{array}$$

Remember that in the end, we seek the specific form of the probability distribution function, which means that we have to solve for the Lagrange multiplier, λ, that appears in both the probability distribution and in the partition function. We can now substitute our specified version of the partition function into the specified versions of the probability distributions, and substitute those into the specified version of our constraint equation. We will do this step by step (and technically in reverse order), starting with substituting the probabilities into the constraint, or Equation (A3) into Equation (A2). This gives us,

(A5) $$\frac{1}{Z}e^{-\lambda }+\frac{2}{Z}e^{-\lambda 2}+\frac{3}{Z}e^{-\lambda 3}=2.$$

Before substituting our partition function, *Z*, Equation (A4), into the constraint equation, Equation (A2), we will first multiply both sides by the partition function, *Z*, to simplify the expression, giving,

(A6) $$e^{-\lambda }+2e^{-\lambda 2}+3e^{-\lambda 3}=2Z.$$

Now we will substitute in the expression for the partition function, *Z*, to arrive at:

(A7) $$e^{-\lambda }+2e^{-\lambda 2}+3e^{-\lambda 3}=2\left(e^{-\lambda }+e^{-\lambda 2}+e^{-\lambda 3}\right).$$

Here we will make an extra substitution, simply for convenience. The equation above is, at heart, a polynomial expression in $e^{-\lambda }$. We can therefore substitute $e^{-\lambda }=x$. Now we will solve for *x*, and at the end we will see what this tells us about λ. Making this substitution, and performing the necessary algebra, we have,

(A8) $$\begin{array}{cc} x+2x^{2}+3x^{3} & =2x+2x^{2}+2x^{3}\\ 0 & =x-x^{3}\\ 0 & =x(1-x^{2}). \end{array}$$

Thus, we have found that the solutions for *x* are x=0, x=1, or x=−1. Recalling that we made the substitution $x=e^{-\lambda }$, this means that we really have $e^{-\lambda }=0$, $e^{-\lambda }=1$, or $e^{-\lambda }=-1$. With these possibilities, we will need to check if one of these possible solutions is the true answer. The other two solutions will prove to give non-sensible probability distributions (complex numbers or non-physical solutions) for the problem that we have specified. To find out which one of the three is correct we must examine the forms of the probability distributions that they yield. We could do this one of two ways. We could use natural logarithm rules to determine the exact possible values of λ, and substitute back into the probability distribution and partition function formulas to find their forms. Or, since both the probability distribution and partition function formulas are functions of $e^{-\lambda }$, we could simply substitute the various known values of 0, −1, or 1 for each occurrence of $e^{-\lambda }$. We will do the latter, as it involves fewer steps.

If $e^{-\lambda }=0$ were the correct answer, then the partition function would take the form of $Z=0^{1}+0^{2}+0^{3}=0$, and the probability distributions would take the form of $p\left(n\right)=0^{n}/Z=0/0$. Dividing by zero is clearly a problem, so $e^{-\lambda }=0$ is not the correct answer. Alternatively, if $e^{-\lambda }=-1$ were the correct answer, then the partition function would take the form of $Z=-1^{1}+{\left(-1\right)}^{2}+{\left(-1\right)}^{3}=-1$, and the probability distribution would take the form of $p\left(n\right)={\left(-1\right)}^{n}/-1=-{\left(-1\right)}^{n}$. This is also a problem, as it means that every probability for rolling an even number would be negative! So $e^{-\lambda }=-1$ is also not the correct answer. This means that $e^{-\lambda }=1$ must be the correct answer. In this case, the partition function takes the form of $Z=1^{1}+1^{2}+1^{3}=3$, and the probability distribution takes the form of $p\left(n\right)=1^{n}/3=1/3$. Remembering that we have a fair die, where fair means equal chances of rolling any number, we see that we have indeed found the correct answer.

### Appendix A.2. A Fair Three-Sided die Constrained by the Standard Deviation

Here we consider a case similar to that above, but with a minor variation. We will constrain the problem with knowledge regarding the standard deviation of the rolls of the die, instead of knowledge regarding the mean value of the rolls of the die. It is still a fair three-sided die, and we will still use the true value one would get for the standard deviation of actual rolls so that we can check our answer against reality at the end. This problem is of interest to us for several reasons. First, it is possible to contrive a situation where all you may know about your data is the standard deviation, and not the mean, yet you still want to determine the corresponding form of the probability distribution functions (perhaps you are reconstructing data from faded paper articles that originally reported both the mean and the standard deviation). Second, as a recent example of the importance of understanding the moments of distributions, Trait-Driver Theory (TDT) in macroecology suggests potential applications using MaxEnt [51,52]. In TDT, higher-order moments of trait distributions are connected with patterns of variability in the local environment and climate. While efforts to link MaxEnt with such environmental variables are ongoing, it may be that constraints based on higher-order moments will prove to be of use in the near future in forecasting how biological organisms will change in response to climate forcings. Finally, this problem is of interest because even though we start with a different constraint equation than Equation (1), we arrive at the same final result that the most fair distribution is the uniform distribution.

To begin we will point out that we will still be using Equations (A3) and (A4) for the specified versions of the probability distributions and the partition function. However, we must express the general form of our new constraint equation differently via the formula for the standard deviation of a distribution of data as,

(A9) $$\sqrt{\sum_{n=1}^{n=N}p\left(n\right){\left[\left(\sum_{m=1}^{m=N}f_{k}\left(m\right)p\left(m\right)\right)-f_{k}\left(n\right)\right]}^{2}}=\sigma_{k}\left(n\right),$$

where $\sigma_{k}\left(n\right)$ is the standard deviation of our data corresponding to the kth constraint. Note that, in Equation (A9), we have assumed that the mean value of our data, $\langle f_{k}\left(m\right)\rangle$ can be expressed as $\sum_{m=1}^{m=N}f_{k}\left(m\right)p\left(m\right)$, which is the same starting expression we have in Equation (1), and thus may be the reason why our approach is still consistent with the central assumptions of MaxEnt.

Continuing on as we did in the previous section, we will substitute everything we know into Equation (A9), starting with the fact that $\sigma_{k}\left(n\right)=\sqrt{2/3}$ for a fair three-sided die (this can be calculated with the definition of the standard deviation and a known, equal probability distribution of all outcomes), and that K=1 for only having one constraint, $f_{k}\left(n\right)=f\left(n\right)=n$, $f_{k}\left(m\right)=f\left(m\right)=m$, and N=M=3. Doing so gives us,

(A10) $$\sqrt{\sum_{n=1}^{n=3}p\left(n\right){\left[\left(\sum_{m=1}^{m=3}mp\left(m\right)\right)-n\right]}^{2}}=\sqrt{\frac{2}{3}}.$$

From here, the steps are quite similar as before, only the algebra is more tedious. We will start with squaring both sides of the equation and writing out the summations explicitly to arrive at,

(A11) $$\begin{array}{cc} p(1) & {\left[p(1)+2p(2)+3p(3)-1\right]}^{2}+\\ p(2) & {\left[p(1)+2p(2)+3p(3)-2\right]}^{2}+\\ p(3) & {\left[p(1)+2p(2)+3p(3)-3\right]}^{2}=\frac{2}{3} \end{array}$$

Substituting $p\left(n\right)=1/Ze^{-\lambda n}=1/Zx^{n}$, where we have also made the substitution of $e^{-\lambda }=x$ for simplification, we have,

(A12) $$\begin{array}{c} \frac{x}{Z}{\left[\frac{x}{Z}+2\frac{x^{2}}{Z}+3\frac{x^{3}}{Z}-1\right]}^{2}+\\ \frac{x^{2}}{Z}{\left[\frac{x}{Z}+2\frac{x^{2}}{Z}+3\frac{x^{3}}{Z}-2\right]}^{2}+\\ \frac{x^{3}}{Z}{\left[\frac{x}{Z}+2\frac{x^{2}}{Z}+3\frac{x^{3}}{Z}-3\right]}^{2}=\frac{2}{3} \end{array}$$

Multiplying both sides of the above expression by $Z^{3}$ to remove all denominators entirely gives,

(A13) $$\begin{array}{c} x{\left[x+2x^{2}+3x^{3}-Z\right]}^{2}+\\ x^{2}{\left[x+2x^{2}+3x^{3}-2Z\right]}^{2}+\\ x^{3}{\left[x+2x^{2}+3x^{3}-3Z\right]}^{2}=\frac{2}{3}Z^{3} \end{array}$$

Now comes a lot of distributing and simplifying of terms. Starting with the left-hand-side, and recalling that $Z=e^{-\lambda }+e^{-\lambda 2}+e^{-\lambda 3}=x+x^{2}+x^{3}$, we arrive at,

(A14) $$x{\left[x^{2}+2x^{3}\right]}^{2}+x^{2}{\left[-x+x^{3}\right]}^{2}+x^{3}{\left[-2x-x^{2}\right]}^{2}=\frac{2}{3}{\left[x+x^{2}+x^{3}\right]}^{3}.$$

Expanding both sides of the expression gives,

(A15) $$x^{4}+5x^{5}+6x^{6}+5x^{7}+x^{8}=\frac{2}{3}x^{3}+2x^{4}+4x^{5}+\frac{14}{3}x^{6}+4x^{7}+2x^{8}+\frac{2}{3}x^{9}.$$

Collecting like terms, we have:

(A16) $$\frac{2}{3}x^{3}+x^{4}-x^{5}-\frac{4}{3}x^{6}-x^{7}+x^{8}+\frac{2}{3}x^{9}=0.$$

Assuming that x=0 is not a solution that we want (recall the discussion earlier regarding this possible solution) we can divide by $x^{3}$ from both sides to yield,

(A17) $$\frac{2}{3}+x-x^{2}-\frac{4}{3}x^{3}-x^{4}+x^{5}+\frac{2}{3}x^{6}=0.$$

We used Wolfram Alpha online (https://www.wolframalpha.com/) to perform the factoring, and find that the above expression can be expressed as,

(A18) $$\frac{1}{3}{\left(x-1\right)}^{2}\left(x+2\right)\left(2x+1\right)\left(x^{2}+x+1\right)=0$$

These terms produce the five following solutions of x=−2, x=−1/2, x=1, and $x=-1/2\pm i\sqrt{3}/2$. As discussed before, the solutions for negative values of *x* are invalid. The solutions $x=-1/2\pm i\sqrt{3}/2$ will result in probabilities with non-real (imaginary) terms, so those will also be disregarded. This leaves us with x=1, which generates the same uniform distribution of p(n)=1/3 as we found in the earlier case for constraining only the mean.

### Appendix A.3. The Gaussian/Normal Distribution, or Using n and n^2^ as “Constraint Functions”

Here we will show that when the “constraint functions” are $f_{k}\left(n\right)=n$ and $f_{k}\left(n\right)=n^{2}$, then the resulting probability distribution is Gaussian, or normal. What it means in an ecological context when we specify these constraint functions may not immediately be clear, but there may be some interesting applications of this derivation, as in reconstructing data when publications specify the variance of a measured distribution, but the underlying data have been lost. With or without a useful applied example, following through with the mathematical derivation lends some insight into the behavior of the equations, so it is useful to proceed for that reason. Without specifying an actual problem, we can not solve explicitly for the Lagrange multipliers. Instead, we will investigate what happens to the mathematical form of the probability distribution in Equation (3) when substituting our constraint information.

As we now have two constraints, K=2, and we will actually be summing over *k* from k=1 to k=2. Additionally, our $f_{k}\left(n\right)$ now have two forms, one being $f_{1}\left(n\right)=n$ and the other being $f_{2}\left(n\right)=n^{2}$. Thus, we could write down the set of constraint equations as,

(A19) $$\begin{array}{cc} \sum_{n=1}^{n=N}np\left(n\right) & =\langle n\rangle \end{array}$$

(A20) $$\begin{array}{cc} \sum_{n=1}^{n=N}n^{2}p\left(n^{2}\right) & =\langle n^{2}\rangle . \end{array}$$

However, for this specific example, we don’t actually know the values of 〈n〉 or $\langle n^{2}\rangle$, so we will go straight to the probability distribution from here. Substituting our constraint functions into the general definition for the probability distribution in Equation (3), we have,

(A21) $$p\left(n\right)=\frac{1}{Z}e^{-(\lambda_{1}n+\lambda_{2}n^{2})}.$$

We can extract more insight from this by re-writing the expression by completing the square. We begin by adding and subtracting by $\lambda_{1}^{2}/4\lambda_{2}^{2}$, in the argument of the exponential,

(A22) $$p\left(n\right)=\frac{1}{Z}e^{-\left(\lambda_{1}n+\lambda_{2}n^{2}+\frac{\lambda_{1}^{2}}{4\lambda_{2}^{2}}-\frac{\lambda_{1}^{2}}{4\lambda_{2}^{2}}\right)}.$$

We can now factor the first three terms in the exponential to arrive at,

(A23) $$p\left(n\right)=\frac{1}{Z}e^{-\left[{\left(\frac{\lambda_{1}}{2\lambda_{2}}+\lambda_{2}n\right)}^{2}-{\left(\frac{\lambda_{1}}{2\lambda_{2}}\right)}^{2}\right]}.$$

This is beginning to look more like the general form of a Gaussian distribution in *n*, aside from the pesky constant term in the argument of the exponential ${\left(\lambda_{1}/2\lambda_{2}\right)}^{2}$. Fortunately, we can use the rules of exponents to rearrange terms and remove the second squared term from the argument in the exponential. We then get,

(A24) $$p\left(n\right)=\frac{e^{{\left(\frac{\lambda_{1}}{2\lambda_{2}}\right)}^{2}}}{Z}e^{-{\left(\frac{\lambda_{1}}{2\lambda_{2}}+\lambda_{2}n\right)}^{2}}.$$

Factoring the $\lambda_{2}$ term in front of *n* gives,

(A25) $$p\left(n\right)=\frac{e^{{\left(\frac{\lambda_{1}}{2\lambda_{2}}\right)}^{2}}}{Z}e^{-\frac{{\left(n+\frac{\lambda_{1}}{2\lambda_{2}^{2}}\right)}^{2}}{1/\lambda_{2}^{2}}}.$$

Now we have arrived at an expression that is more easily recognizable as the general form for a Gaussian distribution in *n*. Comparing this expression to a general Gaussian distribution,

(A26) $$f\left(x|\mu ,\sigma^{2}\right)=\frac{1}{\sqrt{2\sigma^{2}\pi }}e^{-\frac{{\left(x-\mu \right)}^{2}}{2\sigma^{2}}}.$$

We can begin to infer what role the Lagrange multipliers have in this scenario. Noting that the term $\lambda_{1}/2\lambda_{2}^{2}$ sits in the place of the distribution mean μ, we can conclude that the distribution mean is actually given by the specific combination of the Lagrange multipliers of $\lambda_{1}/2\lambda_{2}^{2}$. Similarly, noting that the term $1/\lambda_{2}^{2}$ sits in the place of $2\sigma^{2}$, we can conclude specifically that the standard deviation of the distribution is given by $\sigma =1/\sqrt{2}\lambda_{2}$. Matching the overall prefactors tells us that the combination of the partition function and our left over constant from completing the square combine to normalize the distribution.

### Appendix A.4. The Log-Normal Distribution, Constraining log(n) and log^2^(n)

Everything, or more immediately the results, from the above section can be copied and pasted here but replacing every instance of *n* with logn and $n^{2}$ with $\log^{2}n$ to give us a log-normal distribution.

## Appendix B. Approximations in the Original Version of METE

Two common approximations can be made in METE to simplify the above formulas and provide for limited analytic solutions. In the original presentation of the theory, a number of important assumptions are used. We derive them here because they appear in the Harte (2011) [10], as well as various earlier publications exploring the predictions of METE. The steps and assumptions that are used to derive the original approximations may still be worth testing and exploring, as they are likely sources of measurable error. More recently, a software package in the statistical coding language R [48] called “meteR” [47] has implemented a number of predictions of METE and allows easy comparison of predictions to empirical data. With meteR, it is no longer necessary to make approximations to simplify the core equations of METE. We therefore suggest this as an option for those looking to model empirical data.

### Appendix B.1. Approximation 1: ∑e^−nβ^ ≈ 1/β

The first approximation used throughout Harte (2011) is presented on pages (149–150) simplifies the relationships between the Lagrange multipliers. We define the relationship $\beta =\lambda_{1}+\lambda_{2}$. We consider the first approximation:

(A27) $$\sum e^{-n\beta }\approx 1/\beta .$$

The series $\sum e^{-n\beta }$ is geometric in the variable $e^{-\beta }$. So, as long as $e{}^{-}\beta \neq 0$, then the series can be expressed exactly as,

(A28) $$\sum_{n=1}^{N}e^{-n\beta }=\frac{e^{-\beta }-e^{-\beta (N+1)}}{1-e^{-\beta }}.$$

Assuming that βN>>1, then the second term in the numerator $e^{-\beta (N+1)}\approx 0$, giving,

(A29) $$\sum_{n=1}^{N}e^{-n\beta }\approx \frac{e^{-\beta }}{1-e^{-\beta }}.$$

Furthermore, assuming that β<<1, then $e^{-\beta }\approx 1-\beta$, and we have,

(A30) $$\sum_{n=1}^{N}e^{-n\beta }\approx \frac{1-\beta }{\beta }\approx 1/\beta .$$

In deriving the above expression, we made the simultaneous assumptions that β<<1 and Nβ>>1. This can be expressed in the single expression as 1/N<<β<<1. There are two steps at which these assumptions are employed, thus we could include higher order terms left over from their approximations to get a sense of the resultant order of error associated with their use. It should be clear that this approximation breaks down for small *N*. “Small *N*” will be defined in relation to β; for example, if *N* = 10, then β must take on values greater (or much greater) than 0.1. Because β is defined to be $\lambda_{1}$ + $\lambda_{2}$, we can reference Figure 2 to see how likely a possibility this is for all combinations of $\lambda_{1}$ and $\lambda_{2}$. For single data points, this assumption will always break down, but for large data sets 1/N<<β may indeed be a valid assumption. However, it should also be clear that β<<1 will not hold over most of the parameter space that is possible for ecosystems (see Figure 2). That said, for all of the empirical data sets that have examined $\lambda_{1}$ tends to be small (between 0.001 and 0.1), and β<1, which may be sufficient for these approximations to hold, as advertised, approximately.

The size of β will predominately be influenced by $\lambda_{1}$ in all cases, and it may be the case that the parameter space is more densely populated in the region where $\lambda_{1}$ is small (as we begin to see in Figure 2).

### Appendix B.2. Approximation 2: ∑e^−nβ^/n ≈ log(1/β)

The series $\sum_{n=1}^{N}e^{-n\beta }/n$ is a truncated series expansion for log(1−x). Specifically, for |x|<1,

(A31) $$\log\left(1-x\right)=-\sum_{n=1}^{\infty }\frac{x^{n}}{n}$$

Replacing *x* with $e^{-\beta }$ and breaking the series expansion into two summations, we have,

(A32) $$\log\left(1-e^{-\beta }\right)=-\sum_{n=1}^{N}\frac{e^{-n\beta }}{n}-\sum_{n=N+1}^{\infty }\frac{e^{-n\beta }}{n}$$

Note that this expansion requires $|e^{-\beta }|<1$, which is potentially in conflict with the assumption that β<<1 (an assumption used later in this approximation). This conflict is due to the case that the smaller the value β has, the closer that $e^{\beta }$ is to one. Solving the above expression for the partial series expansion, we have

(A33) $$\sum_{n=1}^{N}\frac{e^{-n\beta }}{n}=-\log\left(1-e^{-\beta }\right)+O\left(\frac{e^{-\beta (N+1)}}{N+1}\right).$$

Here, the “O” represents “order of magnitude of error”, which allows us to quantify the error in the truncation of the series expansion. From our previous Approximation 1, we are simultaneously assuming that Nβ>>1, thus we will drop the $O(e^{-\beta (N+1)}/\left(N+1\right))$ term to arrive at,

(A34) $$\sum_{n=1}^{N}\frac{e^{-n\beta }}{n}\approx -\log\left(1-e^{-\beta }\right).$$

Now, expressing $e^{-\beta }$ as a series we have,

(A35) $$\sum_{n=1}^{N}\frac{e^{-n\beta }}{n}\approx -\log\left(1-\sum_{m=1}^{\infty }\frac{{\left(-\beta \right)}^{m}}{m!}\right).$$

Recalling again the assumption that β<<1, we can truncate the series expansion of $e^{-\beta }$ to $e^{-\beta }\approx 1+\beta$, where we have dropped all terms involving higher powers of β. Doing so, we have

(A36) $$\sum_{n=1}^{N}\frac{e^{-n\beta }}{n}\approx -\log\left(\beta \right)$$

But, −log(β)=0−log(β)=log(1)−log(β)=log(1/β), thus,

(A37) $$\sum_{n=1}^{N}\frac{e^{-n\beta }}{n}\approx \log\left(1/\beta \right).$$

Because we have used the assumption that β<<1 to derive this result, and this assumption may not hold in many cases (see Appendix B.1), both approximations may introduce substantial sources of error in METE’s predictions in some cases, but may otherwise be useful in cases where the user wants to estimate the relative sizes of $\lambda_{1}$ and $\lambda_{2}$. We therefore suggest that calculations involving the Lagrange multipliers be done numerically, without the use of simplifying approximations, with meteR [47] or a similar software.
